# Sex Differences in the Inflammatory Profile in the Brain of Young and Aged Mice

**DOI:** 10.3390/cells12101372

**Published:** 2023-05-12

**Authors:** Brianna Cyr, Juan Pablo de Rivero Vaccari

**Affiliations:** 1 Department of Neurological Surgery and The Miami Project to Cure Paralysis, University of Miami Miller School of Medicine, Miami, FL 33136, USA; bxc205@miami.edu; 2 Center for Cognitive Neuroscience and Aging, University of Miami Miller School of Medicine, Miami, FL 33136, USA

**Keywords:** inflammasome, inflammaging, ASC, IL-1β, caspase-1, sex differences, cytokines, chemokines, brain

## Abstract

Neurodegenerative diseases are a leading cause of death worldwide with no cures identified. Thus, there is a critical need for preventative measures and treatments as the number of patients is expected to increase. Many neurodegenerative diseases have sex-biased prevalence, indicating a need to examine sex differences when investigating prevention and treatment strategies. Inflammation is a key contributor to many neurodegenerative diseases and is a promising target for prevention since inflammation increases with age, which is known as inflammaging. Here, we analyzed the protein expression levels of cytokines, chemokines, and inflammasome signaling proteins in the cortex of young and aged male and female mice. Our results show an increase in caspase-1, interleukin (IL)-1β, apoptosis-associated speck-like protein containing a caspase recruitment domain (ASC), and ASC specks in females compared to males. Additionally, there was an increase in IL-1α, VEGF-A, CCL3, CXCL1, CCL4, CCL17, and CCL22 in aging females and an increase in IL-8, IL-17a, IL-7, LT-α, and CCL22 in aging males. IL-12/IL-23p40, CCL13, and IL-10 were increased in females compared to males but not with age. These results indicate that there are sex differences in cortical inflammaging and provide potential targets to attenuate inflammation to prevent the development of neurodegenerative disease.

## 1. Introduction

Aging is a process common to all humans in which we experience biological changes such as cellular senescence, loss of proteostasis, and chronic inflammation [1]. These changes result in a loss of function and can lead to many age-related diseases, including neurodegenerative diseases. Neurodegenerative diseases lead to morbidity and mortality, a major concern among the increasing elderly population. Furthermore, aged individuals can experience cognitive decline without being diagnosed with a neurodegenerative disease. This age-related cognitive decline includes impairments in learning, memory, motor function, and language [2]. However, modulation of inflammation in the brain has been shown to improve age-related cognitive decline and could be used to prevent and/or delay onset of neurodegenerative diseases [3,4].

Inflammaging, a chronic increased level of inflammation due to old age, has been found to play a role in cognitive decline [3] as well as Alzheimer’s disease (AD) and Parkinson’s disease (PD) [5,6,7]. This chronic inflammation can be measured by the expression levels of cytokines and chemokines. Cytokines and chemokines are signaling proteins secreted by cells for communication. They can be either pro- or anti-inflammatory and can be released by and affect a wide variety of cells. Cytokines and chemokines can have multiple functions in addition to inflammatory signaling that include cell migration and homeostatic functions [8,9]. Cytokines and chemokines are usually examined in cerebrospinal fluid (CSF) or serum in human studies. Increased levels of inflammatory cytokines and chemokines have been found in CSF and serum in cases of age-related cognitive decline as well as neurodegenerative diseases [2,10,11]. Furthermore, a particular inflammatory pathway shown to contribute to inflammaging is the inflammasome [12,13]. The inflammasome is a multiprotein complex consisting of a sensor protein, typically a NOD-like receptor (NLR); an adaptor protein, apoptosis-associated speck-like protein containing a caspase recruitment domain (ASC); and an effector caspase, caspase-1 [14]. The activation of the inflammasome leads to the release of interleukin (IL)-1β and IL-18, two pro-inflammatory signaling molecules [15,16]. The activation of the inflammasome has been found to contribute to inflammaging [12,13] as well as age-related cognitive decline [3] and neurodegenerative disease [17,18,19,20]. Moreover, the inflammasome has also been shown to be a promising target to decrease inflammaging [12] and improve cognitive decline [4,21].

To date, there has been a lack of investigation of inflammatory signaling proteins in the brain during aging as well as between sexes. Previously, we have shown that inflammasome signaling proteins are increased in the cortex and hippocampus of aged male mice [3,12] and that the inflammasome contributes to the inflammatory response in the brain of reproductive senescent females through a mechanism that is partially contributed to by extracellular vesicles containing inflammasome proteins [22]. However, it is known that males and females mount different immune responses throughout their lifetime [23], yet it remains to be fully understood what is the mechanism responsible for the differences in the immune response in the brain between young and aged males and females. There have been some studies of sex differences in inflammatory factors in the brain during aging, but the focus has been specifically on microglia [24,25]. Furthermore, studies investigating age-related cognitive decline have focused more on the hippocampus [3,4] which has been found to be most vulnerable to age-related changes [26]. However, the cortex was also found to be vulnerable to aging and has been shown to have increased inflammation during aging [12,13,24]. Therefore, in this study, we investigate the protein expression levels of the inflammasome, as well as key cytokines and chemokines, in the brain cortex of young and aged male and female mice. Accordingly, we find that there is an increase in the inflammasome adaptor protein ASC and its pathogenic oligomerized form referred to as ASC specks, as well as caspase-1, and IL-1β in aged females compared to males. Furthermore, we find that IL-1α, VEGF-A, CCL3, CXCL1, CCL4, and CCL17 are increased only in aged females, whereas IL-8, IL-17a, IL-7a and LT-α are increased only in aged males. CCL22 was found to be upregulated in aged male and females, whereas IL-12/IL-23p40, CCL13, and IL-10 were found to be increased in females compared to males at the young and aged timepoints, except for IL-10 which was only increased at the aged timepoint.

## 2. Materials and Methods

### 2.1. Animals

All animal procedures were approved by the Animal Care and Use Committee of the University of Miami (protocol 21-192). Animal procedures were carried out according to Guide for the Care and Use of Laboratory Animals (U.S. Public Health). C57BL/6 male and female mice at 3 and 18 months old were sacrificed and the brain cortex was then removed, as described in [12]. Three-month-old female mice were sacrificed during diestrus. Protein lysates were obtained as described in [27] and stored at −80 °C for biochemical analyses. The numbers of animals per group for each analyte have been listed in Table S1.

### 2.2. Immunoblotting

Analyses of inflammasome protein expression were measured by immunoblot analysis, as described in [28]. Briefly, cortical lysates were resolved in 4–20% Criterion TGX Stain-Free precasted gels (Bio-Rad, Hercules, CA, USA), using antibodies (1:1000 dilution) to caspase-1 (Novus Biologicals, Centennial, CO, USA), ASC (Santa Cruz, Dallas, TX, USA), IL-1β (Cell Signaling, Danvers, MA, USA), and β-actin (Sigma Aldric, St Louis, MS, USA). Membranes were imaged using the ChemiDoc Touch Imaging System (Bio-Rad) following chemiluminescence. Quantification of band densities was performed using the UN-SCAN-IT gel 6.3 software (Silk Scientific Corporation, Orem, UT, USA).

### 2.3. Partial Purification of ASC Specks

ASC specks were partially purified as previously described [29]. Briefly, cortical lysates were filtered with 5 μm polyvinylidene difluoride membrane (Millipore, Burlington, MA, USA) at 2000× *g* for 5 min. The filtered supernatant was centrifuged at 5000 rpm for 8 min and the pellet was resuspended in CHAPS buffer. The pyroptosome was pelleted by centrifugation at 5000 rpm for 8 min. The pellet was resuspended in CHAPS buffer and disuccinimidyl suberate (DSS) for 30 min at room temperature to cross-link ASC. An equal volume of 2x Laemmli was added, and samples were immunoblotted for ASC.

### 2.4. Multiplex Assays of Cytokines and Chemokines

Protein concentrations for cytokines and chemokines were analyzed using the V-PLEX Cytokine Panel 1 for GM-CSF, IL-1α, IL-5, IL-7, IL-12/IL-23p40, IL-15, IL-16, IL-17a, LT-α, and VEGF-A, V-PLEX Chemokine Panel 1 for CCL11, CCL26, IL-8, CXCL10, CCL13, CCL22, CCL3, CCL4, and CCL17, and V-PLEX Proinflammatory Panel 1 for IFN-γ, IL-2, IL-4, IL-5, IL-6, IL-10, IL-12p70, CXCL1, and TNF (Meso Scale Discovery, Rockville, MD, USA), as described in [30]. Each panel was completed following the manufacturer’s instructions. Briefly, the provided plate was washed 3 times with the provided wash buffer. A total of 50 µL of diluted sample, controls, or calibrators was loaded into each well of the plate, sealed, and left to incubate at 4 °C overnight. The plate was washed 3 times again with wash buffer and then 25 µL of detection antibody solution was added to each well, sealed, and set to shake for 2 h at room temperature. The plate was washed 3 times with wash buffer, 150 µL of 2x Read Buffer T was added to each well, and only the chemokine panel was incubated for 10 min before analysis. The plate was then analyzed with the MESO Quick Plex SQ120MM and Discovery Bench software.

### 2.5. Statistical Analyses

Following identification and removal of outliers using the ROUT method (Q = 1%), normality was determined with the Shapiro–Wilk test. Comparison between 2 groups was conducted using an unpaired *t*-test or Mann–Whitney test for data that was not normally distributed. Comparison between more than 2 groups was conducted using a two-way ANOVA followed by Tukey’s multiple comparison test. Data that were not normal were normalized using log transformation prior to two-way ANOVA analysis. To calculate percent change, the following formula was used:

$$\frac{aged\;value-young\;value}{young\;value}\times 100$$

Data are presented as mean +/− SEM. The *p*-value of significance was set to less than 0.05 in all tests.

## 3. Results

### 3.1. Inflammasome Proteins Are Increased in the Cortex of Aged Female Mice

**Figure 1:**
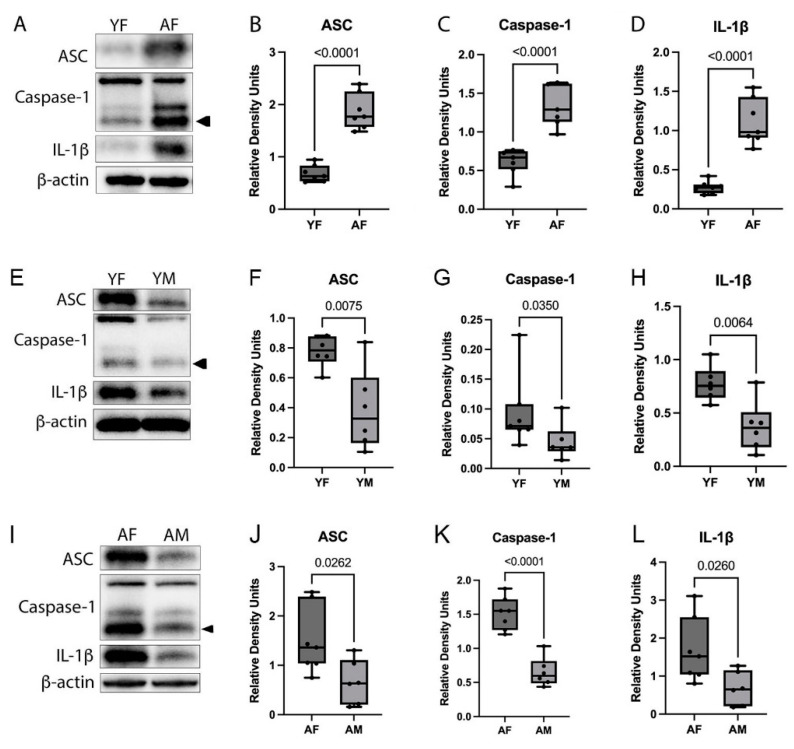
Inflammasome proteins are increased in the cortex of female mice. Mice were sacrificed at young (3 months) and aged (18 months) timepoints. Cortical protein lysates from young and aged males and females were immunoblotted for inflammasome proteins. (**A**) Representative immunoblots for young females vs. aged females and quantification of (**B**) ASC, (**C**) caspase-1, and (**D**) IL-1β. (**E**) Representative immunoblots for young females vs. young males and quantification of (**F**) ASC, (**G**) caspase-1, and (**H**) IL-1β. (**I**) Representative immunoblots for aged females vs. aged males and quantification of (**J**) ASC, (**K**) caspase-1, and (**L**) IL-1β. Data presented as mean +/− SEM. YF: young female, YM: young male, AF: aged female, AM: aged male. N = 6 to 7 per group. β-actin was used as a protein loading control and internal standard.

We have previously shown that inflammasome proteins are increased in the cortex of aged male mice [12]. Here, we aimed to determine if there is a similar pattern of increase in inflammasome proteins in the cortex of aged females and if there is a difference between the levels of inflammasome proteins in males and females. We sacrificed male and female mice at young (3 months) and aged (18 months) timepoints, and the cortex was removed for immunoblot analysis of ASC, caspase-1, and IL-1β. Comparing the inflammasome protein levels between the cortex of young and aged females, we found an increase in ASC, caspase-1, and IL-1β in the cortex of aged mice (Figure 1A–D). In addition, young females compared to young males presented an increase in ASC, caspase-1, and IL-1β in the cortex (Figure 1E–H). Moreover, aged females compared to aged males also presented an increase in ASC, caspase-1, and IL-1β in the cortex (Figure 1I–L). Although females presented greater expression of inflammasome proteins, a calculation of percent change from young to aged for males and females revealed that the rate of increase in these proteins from young to aged was similar (Supplemental Figure S1). Thus, while females may have greater levels of inflammasome activation, the rate of inflammasome activation increase due to inflammaging is the same between males and females.

### 3.2. ASC Specks Are Increased in the Cortex of Aged Female Mice

**Figure 2:**
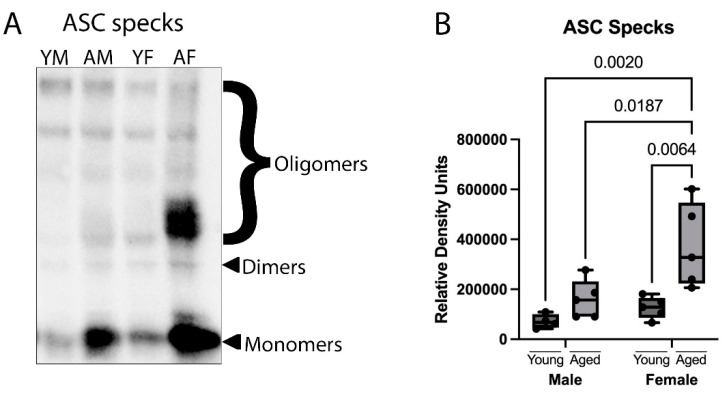
ASC specks are increased in the cortex of aged female mice. Cortical protein lysates from young (3 months) and aged (18 months) male and female mice underwent partial purification of the pyroptosome. (**A**) Representative immunoblot of cortical lysate of young and aged male and female mice blotted for ASC. (**B**) Quantification of ASC specks. Data presented as mean +/− SEM. YF: young female, YM: young male, AF: aged female, AM: aged male. N = 4 to 5 per group.

ASC specks can be released due to pyroptosis, and after release, they maintain their ability to process IL-1β in the extracellular space [31]. To determine whether there is an increase in ASC speck formation in the cortex of aged mice, ASC specks were isolated via the partial purification of the pyroptosome from cortical lysates and immunoblotted for ASC oligomers (Figure 2A). Accordingly, there was an increase in ASC specks in both aged male and female mice; however, only female mice showed a significant increase (Figure 2B). Additionally, the levels of ASC specks in the aged female cortex were significantly greater than in the aged male cortex. Together, these findings indicate that these prion-like assemblies are significantly elevated in the brain of aged female mice.

### 3.3. Cytokines Are Differentially Upregulated in the Cortex of Aging Mice

**Figure 3:**
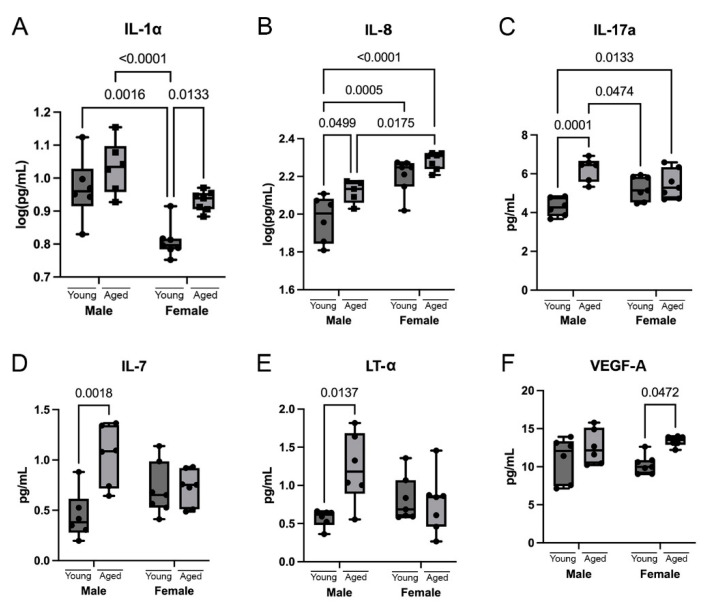
Sex and age differences in cytokine levels in the cortex of mice. Cortical protein lysates from young (3 months) and aged (18 months) male and female mice were analyzed for cytokine levels using multiplex assays. The cytokines that show a significant increase in aging are (**A**) IL-1α, (**B**) IL-8, (**C**) IL-17a, (**D**) IL-7, (**E**) LT-α, and (**F**) VEGF-A. Data presented as mean +/− SEM. N = 5 to 7 per group.

**Figure 4:**
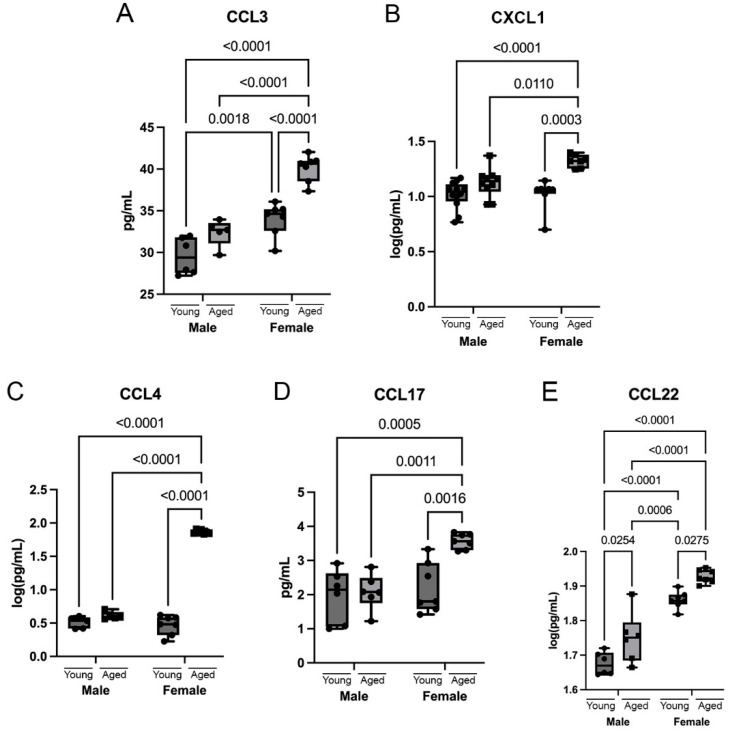
Sex and age differences in chemokine levels in the cortex of mice. Cortical protein lysates from young (3 months) and aged (18 months) male and female mice were analyzed for chemokine levels using multiplex assays. The chemokines that show a significant increase in aging are (**A**) CCL3, (**B**) CXCL1, (**C**) CCL4, (**D**) CCL17 and (**E**) CCL22. Data presented as mean +/− SEM. N = 5 to 12 per group.

Cytokines, such as IL-1α, IL-1β, and IL-17, have been found to be upregulated in the hippocampus of aged mice [32]. Here, we determined the levels of several cytokines in the cortex of young and aged male and female mice. IL-1α levels were increased during aging in the cortex of female mice but not in male mice (Figure 3A). Interestingly, IL-1α levels were greater in young males compared to young females. IL-8 levels were increased in the cortex of aged males but not in females (Figure 3B). However, IL-8 levels were greater in females than males at both young and aged timepoints. IL-17a levels were increased in aged males but not aged females (Figure 3C). The levels of IL-17a were not significantly different between males and females at the young or aged timepoints. IL-7 levels were increased in the brain of aged males but not females (Figure 3D), and there were no differences in the levels of IL-7 between sexes at either the young or aged timepoints. Lymphotoxin (LT)-α levels were increased in aged males but not aged females (Figure 3E). There were no differences in the levels of LT-α between sexes at the young and aged timepoints. Finally, there was an increase in the levels of vascular endothelial growth factor (VEGF)-A in aged females but not males (Figure 4F). Similarly, there were no differences in the levels of VEGF-A between sexes at the young and aged timepoints. Additionally, we found that the cytokines IL-2, IL-4, IL-5, IL-6, IL-12p70, IL-15, IL-16, GM-CSF, TNF, and IFN-γ did not show any significant differences during aging or between sexes (Supplementary Figure S2). These findings indicate an inflammatory response that is mediated by cytokines that differs as a result of age and sex.

### 3.4. Chemokines Are Differentially Upregulated in the Cortex of Aging Mice

Chemokines, such as CCL3, CCL4, and CXCL1, have been found to be upregulated in the hippocampus of aged mice [32]. Here, we determined the levels of various chemokines in the cortex of young and aged male and female mice. The protein levels of CCL3 were increased in aged females but not in males (Figure 4A). CCL3 levels were also increased in females compared to males at both young and aged timepoints. The protein levels of CXCL1 were also increased in aged females but not in males (Figure 4B). CXCL1 levels were increased in females compared to males only at the aged timepoint. The protein levels of CCL4 were increased in aged females but not in males (Figure 4C). In addition, there was also an increase in CCL4 levels in females compared to males only at the aged timepoint. The protein levels of CCL17 were increased in aged females but not in males (Figure 4D). Similarly, CCL17 levels were increased in females only at the aged timepoint compared to males. Lastly, there was an increase in the protein levels of CCL22 in both aged males and females (Figure 4E). However, CCL22 levels were still higher in females at the young and aged timepoints when compared to males. Additionally, we investigated the chemokines CCL11, CCL26, and CXCL10, which did not show any significant differences in aging or between the sexes (Supplementary Figure S3). These findings indicate an inflammatory response that is mediated by chemokines that differs as a result of age and sex.

### 3.5. Sex Differences in Cytokines and Chemokines

**Figure 5:**
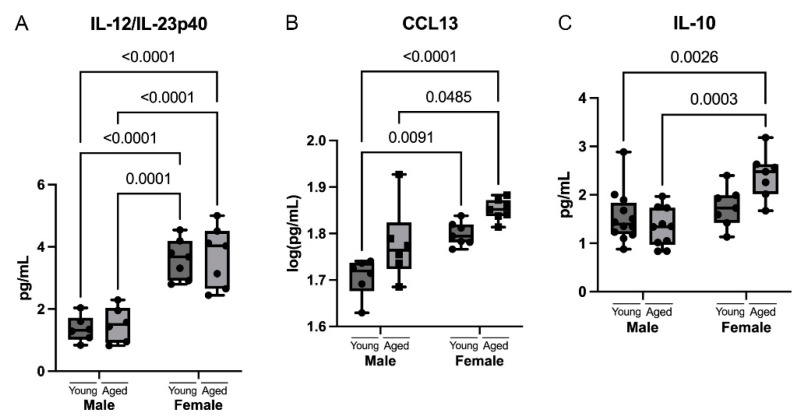
Sex differences in cytokines and chemokines in the cortex of mice. Cortical protein lysates from young (3 months) and aged (18 months) male and female mice were analyzed for cytokine and chemokine levels using multiplex assays. The cytokines and chemokines that show a significant difference between sexes regardless of age are (**A**) IL-12/IL-23p40, (**B**) CCL13, and (**C**) IL-10. Data presented as mean +/− SEM. N = 6 to 12 per group.

Cytokine and chemokine regulation can differ between males and females of the same age [23,25,33]. Our analysis showed that there was an increased upregulation of IL-12/IL-23p40 and CCL13 in females compared to males at both the young and aged timepoints, and there was no significant increase with age (Figure 5A,B). Additionally, we also found that IL-10 was increased in aged females compared to aged males and had no significant increase with age (Figure 5C), indicating that IL-12/IL-23p40, CCL13, and IL-10 are differentially regulated only in males versus females, but not as a result of age.

## 4. Discussion

Inflammaging is a chronic inflammatory condition that can lead to neurodegenerative disease and age-related cognitive decline [3,6,7]. Previous studies have shown sex differences in cytokines and chemokines in different areas of the brain and in certain cell types due to aging [24,32]. Sex differences have also been found in inflammasome proteins in different disease models [22,34,35]. However, there is still a lack of research regarding the differences in male versus female inflammaging. To the best of our knowledge, this is the first study to report sex and age differences in the expression levels of cytokines, chemokines, and inflammasome proteins in the cortex of young and aged male and female mice. Here, we present data indicating that male and female mice experience cortical brain inflammaging, but the inflammatory factors that contribute to inflammaging in sex and at different ages (3 months versus 18 months) are not the same across groups. Overall, our results indicate key inflammatory factors that lead to age-related cognitive decline. Additionally, these findings provide important information regarding why there are significant sex differences in the prevalence of different neurodegenerative diseases and provide potential targets for treatment and/or disease prevention.

We have previously shown the role of the inflammasome and the effects of blocking inflammasome activation in the cortex of aged male rodents [3,12]. In this study, we extended on the previous findings and investigated the levels of inflammasome complex proteins as well as an array of key inflammatory cytokines and chemokines in the cortex of young and aged male and female mice. Here, we found that females also experience an increase in inflammasome proteins in the cortex during old age and that these levels are significantly higher than in aged males. In addition, we found that the expression levels of different cytokines and chemokines in the cortex of mice differ between males and females.

In this study, we have found that the inflammasome proteins caspase-1, ASC, IL-1β, and ASC specks are elevated in the cortex of aged females compared to young females and aged males. Increased levels of inflammasome proteins have been linked to age-related cognitive decline as well as AD and PD [3,7,19,21,36]. Our data corroborate another study which has shown that reproductive senescent female rats experience an increase in inflammasome protein levels in the brain [22]. However, there is still a lack of research regarding the differences in the levels of inflammasome proteins in males versus females during normal aging in the brain.

Furthermore, here, we found that levels of IL-1α, VEGF-A, CCL3, CXCL1, CCL4, and CCL17 are only increased in aged females. IL-1α has many of the same functions as IL-1β; however, IL-1α is not directly involved downstream of the inflammasome pathway [37]. IL-1α has been found to increase with age in the brain; however, it was unknown whether IL-1α levels differed in males versus females [32]. VEGF-A is known to function in angiogenesis and neuroprotection. It has been implicated in many neurodegenerative diseases, mainly AD and PD [38,39]. The literature on VEGF-A is conflicting, with some studies indicating that increases in VEGF-A are associated with AD [40], while other studies have actually shown that a decrease in VEGF-A is associated with AD pathology [41]. Although here we report a significant increase in VEGF-A only in aged females, there is a lack of research into the effects of sex differences on VEGF-A, especially since it has been previously demonstrated that VEGF-A functions differently in males versus females [42]. CCL3 and CCL4 are closely related, binding to the same receptor, namely CCR5. CCL3 and CCL4, along with CXCL1, have been found to be upregulated in the brain due to age and AD [32,43]. A previous study investigating sex differences for CCL4 in the aging brain also found similar results to our study. Accordingly, the increased levels of CCL4 in aged females was highly significant compared to all other groups [24]. CCL17 is known to have inflammatory as well as regulatory functions and has been implicated in the pathogenesis of multiple sclerosis (MS) [44]. However, the levels of CCL17 in normal aging or other neurodegenerative diseases have not been previously investigated.

Protein levels of IL-8, IL-17a, IL-7, and LT-α are increased only in aged males. IL-8 plays a role in inflammation as well as angiogenesis [45]. IL-8 increases with age [46] and IL-8 upregulation in the brain has been associated with AD [43]; however, there was no report of sex differences in these studies. IL-17a has been found to contribute to many inflammatory conditions [47]. Levels of IL-17a have been found to be increased in many neurodegenerative diseases, including AD, PD, MS, and amyotrophic lateral sclerosis (ALS) [48]. Although there is extensive research on IL-17a in neurodegeneration, and sex differences are known to exist in the periphery [23], it is unknown if there are sex differences in normal brain aging or neurodegeneration. IL-7 is known for B- and T-cell regulation but has been shown to function as a growth factor in neural development [49]. IL-7 is mainly involved in peripheral inflammatory conditions [50], yet there has been limited research on IL-7 in the brain. LT-α is in the tumor necrosis factor (TNF) superfamily of cytokines but is not as well studied as TNF. Peripherally, it has been shown to induce cell death and inflammatory signaling as well as lymphoid tissue organogenesis [51,52], and the levels of LT-α in the brain or during aging have not been well studied.

CCL22 was the only cytokine or chemokine found to be upregulated in aged males and aged females, suggesting a unique contribution of CCL22 to inflammaging that is sex independent. However, the levels of CCL22 were significantly higher in females than males at the young and aged timepoints. CCL22 binds to the same receptor as CCL17. Thus, it shares a similar function [53]. CCL22 has been found to be implicated in MS [53,54]; however, to the best of our knowledge, there have been no studies indicating CCL22 elevation in age-related neurodegenerative diseases. One previous study has investigated the levels of CCL22 in the hippocampus and cortex microglia of neonatal and adult rats. That study found CCL22 to be significantly upregulated in females compared to males at P4, but the effect was reversed at P60 [33]. Hence, there is a need to investigate sex and age differences in CCL22 in different brain regions and cell types at different developmental stages and in adulthood.

Overall, we found more cytokines and chemokines increased due to aging in females compared to males, and in most cases, females presented higher levels in the cortex than their age-matched male counterparts. This suggests that females experience inflammaging earlier than males and/or have more severe inflammaging than males. Indeed, it has been reported that females have higher amounts of inflammatory factors when compared to males [24,55]. It is theorized that females experience this greater inflammation, particularly in aging, due to the decrease in hormonal signaling following menopause [56]. Furthermore, a previous study on murine reproductive senescence has shown that extracellular vesicles carrying inflammasome proteins also contribute to brain inflammaging in aged females [22].

In this study, there were two cytokines and one chemokine (IL-12/IL-23p40, IL-10, and CCL13) that did not have a significant increase as a result of age but did have a significant difference between sexes. IL-12/IL-23p40 is a p40 subunit of both IL-12 and IL-23 but may have biological activity on its own [57]. Our results show female mice showed significantly higher levels of IL-12/IL-23p40 at both young and aged timepoints compared to males. However, there is no significant change, nor a trend, during aging in either males or females. Previous studies reported IL-12/IL-23p40 to be elevated in the serum in old age and in the ventral hippocampus due to aging [32,58]. In the ventral hippocampus, it appeared that the levels of IL-12/IL-23p40 were greater in females; however, the study was not powered to examine sex differences. IL-10 is an anti-inflammatory cytokine that helps to resolve inflammation. Our results show that there are increased levels of IL-10 in aged females compared to aged males, and we found no significant increase in IL-10 due to age, but there was a trend of higher levels only in females. Another study has also found IL-10 to be increased in females compared to males in the cortex and hippocampus [33]. IL-10 has been found to be elevated in both AD and PD, where it is thought to play a neuroprotective role [59]. CCL13 is a chemokine that attracts pro-inflammatory cells but is also expressed under homeostasis in a number of peripheral tissues [60]. Our results show that there is a trend of increased levels in aged males and females, but females presented significantly higher levels of CCL13 compared to males at the young and aged timepoints. CCL13 has been found to be implicated in MS [60]; however, there is limited research on CCL13 in the brain and what its function might be in the brain.

Furthermore, it is important to note that cytokines and chemokines can have a variety of effects, and it is not well understood whether these signaling molecules have the same or different functions in the central nervous system (CNS). It is possible that in the CNS, cytokines and chemokines can have different roles that do not apply to the periphery. For example, CCL22 has been found to be involved in temperature regulation and neuronal calcium signaling [61,62] and IL-7 can serve as a neuronal growth factor during development [49]. Similarly, we have found differential levels of these proteins in the cerebral circulation and the peripheral circulation after stroke in humans [30]. Thus, it is prudent to investigate if there are any additional effects that cytokines and chemokines may have in the CNS that may differ from the periphery.

Many cytokines and chemokines presented a trend towards higher protein levels due to aging; however, these were not significant. Interestingly two key inflammatory cytokines that are often seen in inflammaging, IL-6 and TNF, were not found to be significantly increased but were trending to be increased with age. The age of the aged mice (18 months) for this study corresponds to approximately 56 years of age in humans [63]. This is prior to the age of onset for most neurodegenerative diseases, with the typical age of onset for AD being 65 and older [64], and for PD being 70 and older [65]. Thus, this study provides insight into early inflammaging, and it is expected that cytokines and chemokines that present a trend towards higher levels will become significant in older mice.

To build upon this work, future studies should investigate the expression levels of the analytes examined here during middle age, reproductive senescence, and older age timepoints to determine how protein levels change across the lifespan and affect cognitive function. This will provide further insight into how cytokine and chemokine levels fluctuate with age and may reveal more relevant targets for age-related cognitive decline. Further studies on the levels of inflammatory protein changes across the estrous cycle are also required since it has been shown that fluctuations in inflammatory proteins occur in reproductive organs but no studies have been conducted in the brain [66]. Future studies should also investigate sex differences in aged mice compared to mouse models of neurodegenerative diseases such as AD and PD, to determine differences in the neuroinflammatory profile of the brain due to inflammaging versus neurodegenerative disease. Furthermore, it would be helpful to examine the levels of these proteins in the CSF of the animals as well as to determine if there is a correlation between the levels of inflammatory proteins between the CSF and the cortical lysate. This has the potential to make the results more relevant for translational studies as most cytokine measurements in humans will come from CSF or serum.

In conclusion, this study provides relevant inflammatory targets by determining protein expression patterns in which cytokines and chemokines are increased due to age. In addition, this study shines light onto inflammatory targets that can be examined to better understand why males or females are more likely to be diagnosed with certain neurodegenerative diseases.

## Data Availability

The data presented in this study are available on request from the corresponding author.

## Supplementary Materials

The following supporting information can be downloaded at: https://www.mdpi.com/article/10.3390/cells12101372/s1, Figure S1: Inflammaging of inflammasome proteins occurs at the same rate in male and female mice. Figure S2: Cytokines that did not differ between sexes or between different ages. Figure S3: Chemokines that did not differ between sexes or with age. Table S1: Number of mice used per analyte.
