# Supplementary material for: Sex Differences in the Inflammatory Profile in the Brain of Young and Aged Mice
Source: Cells. 2023 May 12;12(10):1372. doi: 10.3390/cells12101372 (PMC10216304; doi:10.3390/cells12101372)
Supplement: Supplementary file 1 [file cells-12-01372-s001.zip › cells-2376665-supplementary.pdf]

Figure S1

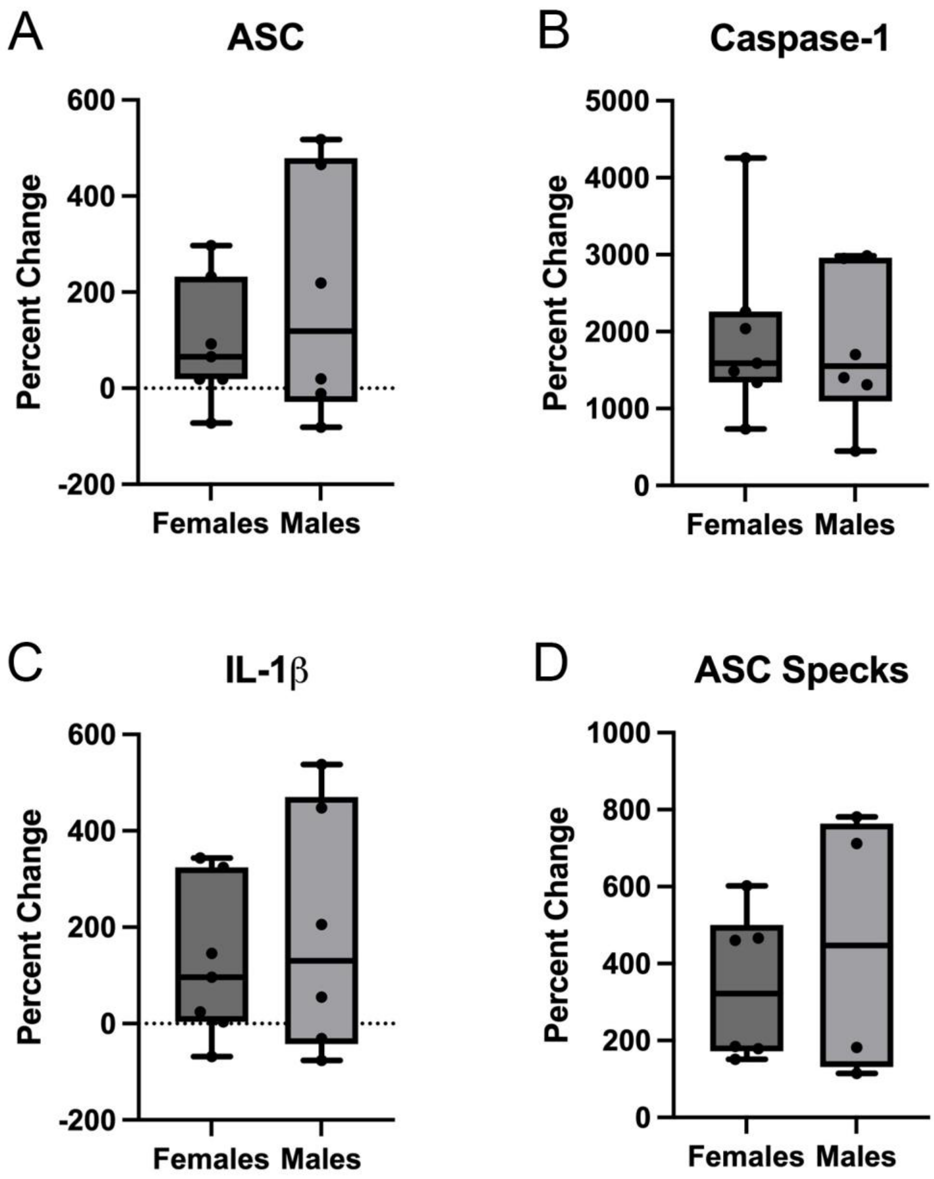

A
ASC
600
400
200
0
-200
Percent Change
Females
Males
B
Caspase-1
5000
4000
3000
2000
1000
0
Percent Change
Females
Males
C
IL-1β
600
400
200
0
-200
Percent Change
Females
Males
D
ASC Specks
1000
800
600
400
200
0
Percent Change
Females
Males

Figure S2

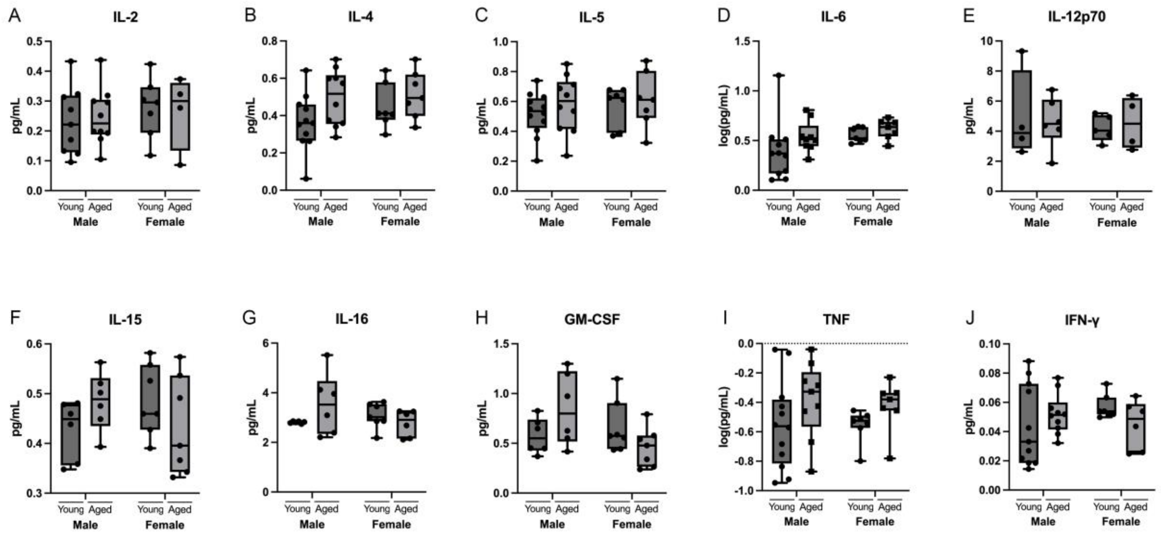

Figure S3

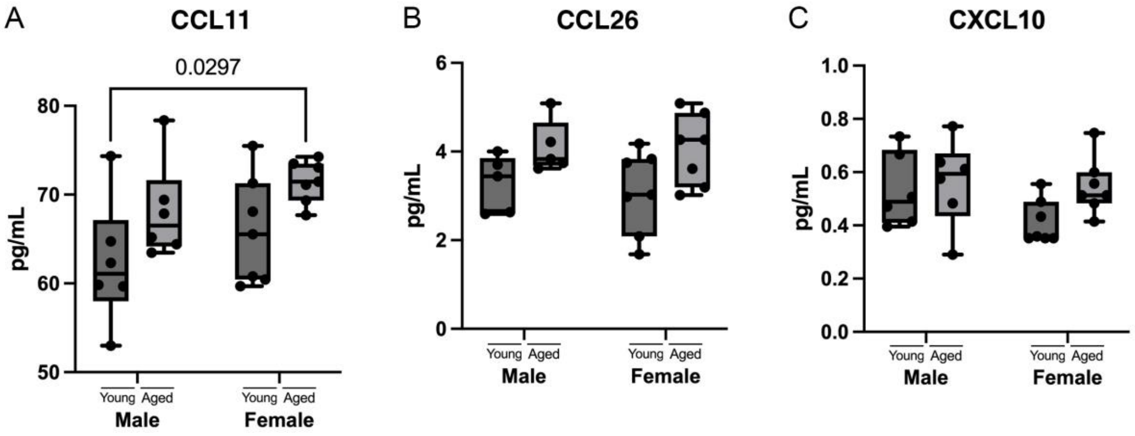

Table S1. Number of mice used per analyte.

	Young Female	Aged Female	Young Male	Aged Male
ASC	n = 7 (YF vs. AF)<br>n = 6 (YF vs. YM)	n = 7	n = 6	n = 6
Caspase-1	n = 7	n = 7	n = 6	n = 6
IL-1β	n = 7 (YF vs. AF)<br>n = 6 (YF vs. YM)	n = 7	n = 6	n = 6
ASC specks	n = 5	n = 5	n = 4	n = 5
GM-CSF	n = 7	n = 7	n = 6	n = 6
IL-1α	n = 7	n = 7	n = 6	n = 6
IL-5	n = 7	n = 7	n = 6	n = 6
IL-7	n = 7	n = 7	n = 6	n = 6
IL-12/IL-23p40	n = 7	n = 7	n = 6	n = 6
IL-15	n = 7	n = 7	n = 6	n = 6
IL-16	n = 7	n = 6	n = 6	n = 6
IL-17a	n = 7	n = 7	n = 6	n = 6
LT-α	n = 7	n = 7	n = 6	n = 6
VEGF-A	n = 7	n = 6	n = 6	n = 6
CCL11	n = 7	n = 7	n = 6	n = 6
CCL26	n = 7	n = 7	n = 5	n = 5
IL-8	n = 7	n = 7	n = 6	n = 5
CXCL10	n = 7	n = 7	n = 6	n = 6
CCL13	n = 7	n = 7	n = 6	n = 6
CCL22	n = 7	n = 7	n = 6	n = 6
CCL3	n = 7	n = 7	n = 6	n = 5
CCL4	n = 7	n = 7	n = 5	n = 5
CCL17	n = 7	n = 7	n = 6	n = 6
IFN-γ	n = 7	n = 6	n = 11	n = 10
IL-2	n = 7	n = 4	n = 9	n = 10
IL-4	n = 7	n = 7	n = 11	n = 10
IL-5	n = 7	n = 7	n = 12	n = 10
IL-6	n = 7	n = 7	n = 11	n = 10
IL-10	n = 7	n = 7	n = 12	n = 10
IL-12p70	n = 5	n = 4	n = 4	n = 6
CXCL1	n = 7	n = 7	n = 12	n = 10
TNF	n = 7	n = 7	n = 12	n = 9
